# Reporting of health estimates prior to GATHER: a scoping review

**DOI:** 10.1080/16549716.2017.1267958

**Published:** 2017-05-22

**Authors:** Mia Cokljat, James Henderson, Angus Paterson, Igor Rudan, Gretchen A. Stevens

**Affiliations:** ^a^ Centre for Global Health Research, Usher Institute for Population Health Sciences and Informatics, University of Edinburgh, Edinburgh, UK; ^b^ Department of Information, Evidence and Research, World Health Organization, Geneva, Switzerland

**Keywords:** Reporting guideline, GATHER Health estimate, Health status, Risk factors

## Abstract

**Background**: Generating estimates of health indicators at the global, regional, and country levels is increasingly in demand in order to meet reporting requirements for global and country targets, such as the sustainable development goals (SDGs). However, such estimates are sensitive to availability of input data, underlying analytic assumptions, variability in statistical techniques, and often have important limitations. From a user perspective, there is often a lack of transparency and replicability. In order to define best practices in reporting data and methods used to calculate health estimates, the Guidelines for Accurate and Transparent Health Estimates Reporting (GATHER) working group developed a minimum checklist of 18 items that must be reported within each study publishing health estimates, so that users may make an assessment of the quality of the estimate.

**Objective**: We conducted a scoping review to assess the state of reporting amongst a cross-sectional sample of studies published prior to the publication of GATHER.

**Methods**: We generated a sample of UN reports and journal articles through a combination of a Medline search and hand-searching published health estimates. From these studies we extracted the percentage of studies correctly reporting each item on the checklist, the proportion of items reported per study (the GATHER performance score), and how this score varied depending on study type.

**Results**: The average proportion of items reported per study was 0.47, and the poorest-performing items related to documentation and availability of input data, availability of the statistical code used and the subsequent output data, and a complete detailed description of all the steps of the data analysis.

**Conclusions**: Methods for health estimates are not currently fully reported, and the implementation of the GATHER guidelines will improve the availability of information required to make an assessment of study quality.

## Background

Data on health indicators, for example, child mortality, life expectancy, or the prevalence of obesity, are needed to set policy priorities, allocate funding, and evaluate programmes [1]. However, measurements of these indicators may differ in methodology, and are often not available for all populations and time periods. For this reason, statistical or mathematical models are often employed in order to generate estimates of key quantities of interest from the available health data. Although these statistical methods can generate reliable estimates, they are sensitive to underlying analytic assumptions and often have important limitations. In addition, the methods are complex and difficult to explain to users who may not have the same level of statistical expertise.

Despite these limitations, there is growing reliance on estimates of health indicators for tracking progress towards global and regional goals and targets. Health estimates allow for objective and comparative analyses of health and disease worldwide, and may be used to guide decisions on global health policies, priorities, and resource allocation. Therefore, it is paramount that the methods and limitations of these estimates, both in terms of underlying input data and of analysis, be reported so that users may understand how they have been derived, and their relevance, appropriateness, and fitness for purpose [2–5]. In addition, data and methods should be transparent so that other researchers can build upon published research to advance the science of health estimation [6,7].

At World Health Organization (WHO) expert meetings of February and December 2013, participants agreed that transparency of methods was inadequate and that reporting guidelines were needed [8]. In response, the WHO convened the working group on Guidelines for Accurate and Transparent Health Estimates Reporting (GATHER) and tasked it to develop reporting guidelines for global health estimates. The output of the working group, the GATHER guidelines, is a checklist of 18 items to be reported in every publication of health estimates [4,5]. The presence of this crucial methodological information should allow both expert and non-expert audiences to make an assessment of the quality of the methods and the resulting estimates.

In this study, we aim to establish the state of reporting prior to the publication of the GATHER guidelines. We do so through a scoping review in which we quantitatively assess whether a cross-sectional sample of studies are reported in compliance with the GATHER checklist.

## Methods

Our search strategy was designed to obtain a sample of recent studies that fall within the scope of GATHER, rather than a comprehensive inventory of health estimates publications. Because many health estimates are published by United Nations (UN) agencies, specifically the WHO, United Nations Population Division, and the United Nations Children’s Fund (UNICEF), we developed a search strategy that would cover both journal articles and UN reports.

### Scope

Any study falling within the scope of GATHER was eligible for inclusion [4,5]. GATHER defines best reporting practices for studies that report population-level estimates of a health indicator measuring either health status or a subset of proximal health determinants. To fall within GATHER’s scope, an estimate must be calculated by statistically synthesizing data from multiple data sources to generate quantitative health estimates that vary by time or by geography.

### Development of a sample of target articles

Because studies that fall within the scope of GATHER cover a diverse range of health topics and populations studied, we *a priori* identified a set of target journal articles known to the authors to be within the scope of GATHER. These target articles were subsequently used to guide the development of search terms which would retrieve articles reporting health estimates. We selected the target articles to cover the health-related millennium development goals (MDGs) and the top 10 causes of global Disability-Adjusted Life Years (DALYs), as reported by the WHO 2012 report, to represent health status [9]; and the top 5 global risk factors in 2010 as reported by the Global Burden of Disease 2013 study to represent health determinants [10]. The target articles also ranged in geographic scope from national to global. Thus, these articles covered a range of current research fields. The target articles are listed in Supplementary material 1.

### Search strategy: journal articles

The literature search was conducted using Medline, and included articles published in English between January 2010 and July 2015. We sought a balance between identifying all of the articles in our sample of target papers (sensitivity), and the proportion of articles retrieved that were within scope (specificity). Therefore, the development of the search terms consisted of using different combinations of MeSh (medical subject headings) terms on specific health topics and epidemiological limits; these combinations were tested against the pool of target articles, and a percentage of articles in the pool and identified by the search was calculated. The final search terms are shown in Supplementary material 2.

### Determination of eligibility

The following inclusion criteria were applied:

Study type

We included studies that synthesize data from multiple data sources, in order to generate quantitative health estimates of health status and determinants that vary by time or geographic population.

Geographic coverage

Articles reporting health estimates at the global, regional, national, or subnational levels were included.

Types of health indicators

Articles estimating indicators of health status (e.g. total and cause-specific mortality, incidence and prevalence of diseases, injuries, disabilities, hospital admissions, and diseases attributable to a cause) and health determinants (risk factors to health, including health behaviours such as tobacco and alcohol use, and health exposures such as obesity) were included. Note that any health estimate within scope was included, not only those covering the specific topics of the target articles.

We excluded articles reporting indicators from a single data source, including data sources that cover multiple years or geographic populations. We considered articles that combined one data source with population denominators to be a report on a single data source, and therefore excluded them. We also excluded articles reporting service coverage or health systems indicators [11] and articles where full texts and/or supplementary material could not be obtained.

**Figure 1.**
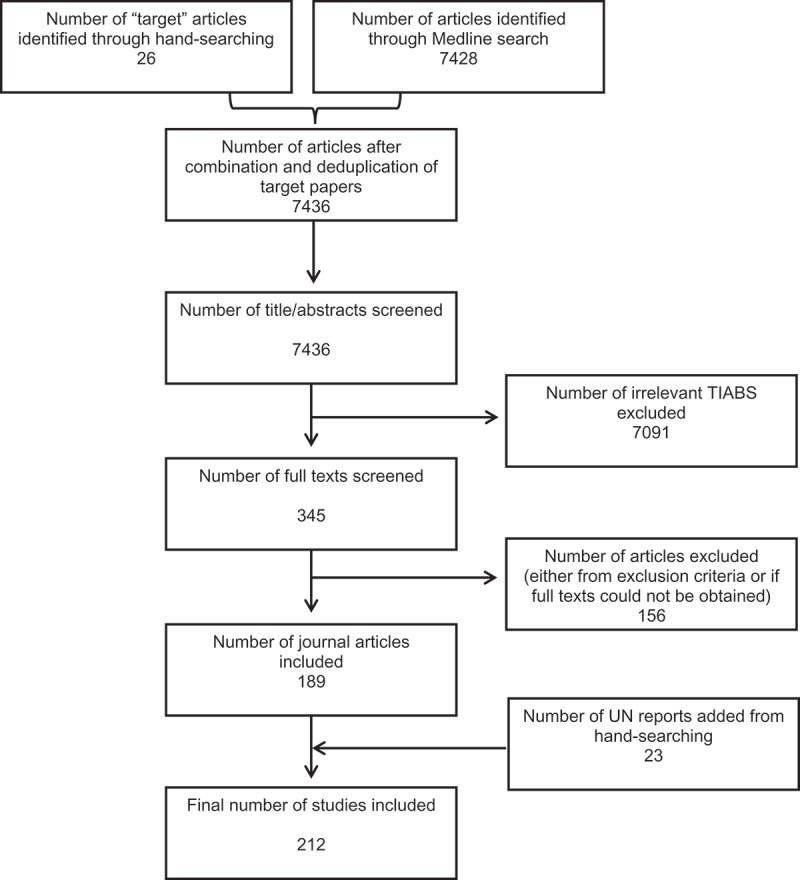
Flowchart showing the selection process of eligible studies.

The title/abstract review was divided between two independent reviewers. In order to ensure reproducibility, training on the GATHER inclusion/exclusion criteria for title/abstracts (TIABs) was performed on 200 randomly selected articles from the search, after which Cohen’s kappa was calculated for inter-observer agreement on a further 200 randomly selected articles. The remaining articles were then split equally between two independent reviewers for a full TIAB review, and irrelevant TIABs excluded. After this, full texts were obtained and a similar initial training process was undertaken using the included TIABs remaining from the 400 randomly selected articles. Once acceptable inter-observer agreement was achieved, the remaining full texts were divided equally amongst the two reviewers (MC and AP) and any irrelevant full texts were excluded. Finally, the inclusions were then cross-checked by the other reviewer and any disputes were settled by a third reviewer (GAS). Endnote was used to store the retrieved references from Medline. The final sample of journal articles included the articles identified through the systematic search, and remaining articles from the sample of target articles that were not selected by the search (Figure 1).

### Search strategy: UN reports

UN reports are not indexed by Medline. Thus, a sample of reports was selected by hand-searching UN websites for reports covering the same topics of interest used to select the target articles for the Medline search (i.e. health-related MDGs, top causes of burden of disease, and leading risk factors). Therefore, the final sample of studies consisted of the pool of target articles, the articles generated by the Medline search, and hand-selected UN reports.

### Data extraction

Full-text articles and any supplementary materials were obtained for all eligible articles and reports. The following descriptive information was extracted: the type of estimate being reported (status or determinant), journal of publication and its impact factor (if applicable), year of publication, country of origin according to the corresponding author’s address, and geographical population coverage (e.g. national, global).

Before the studies were assessed for compliance with the GATHER checklist, the checklist items with multiple parts were divided into separate components (items 3, 4, 5, and 13; see Table 1). For item 5, a table of seven characteristics for each input data source is required to fulfil the criteria. Often studies report some of these characteristics, but rarely all seven; therefore, we assessed whether studies report a minimum of four characteristics (5a), and whether they report the complete set of seven (5b). Additionally, item 6 (‘identify and describe any categories of input data that have potentially important biases’) was excluded from assessment in this scoping review. This is because the reviewers did not have adequate expertise in the wide range of health indicators calculated to assess whether potential biases of the input data were described.

**Table 1.** GATHER checklist of information that each study making global health estimates is required to report, including information on how reporting was assessed in the scoping review. Divisions of some multi-part reporting items resulted in *n* = 21 number of reporting items for the purpose of the scoping review.

Item #	Checklist item	Comments on application in the scoping review
**Objectives and funding**		
1	Define the indicator(s), populations (including age, sex, and geographic entities), and time period(s) for which estimates were made.	None
2	List the funding sources for the work.	None
**Data inputs**		
**For all data inputs from multiple sources that are synthesized as part of the study:**		
3	Describe how the data were identified and how the data were accessed.	3a – describe how the data were identified 3b – describe how the data were accessed
4	Specify the inclusion and exclusion criteria. Identify all ad-hoc exclusions.	4a – specify inclusion and exclusion criteria, or the database from which the data were retrieved 4b – identify ad-hoc exclusions, may score NOT RELEVANT
5	Provide information on all included data sources and their main characteristics. For each data source used, report reference information or contact name/institution, population represented, data collection method, year(s) of data collection, sex and age range, diagnostic criteria or measurement method, and sample size, as relevant.	5a – provide at least 4 of the listed characteristics for each data source 5b – provide all 7 characteristics
6	Identify and describe any categories of input data that have potentially important biases (e.g. based on characteristics listed in item 5).	Not assessed as the reviewers did not have adequate expertise to make an assessment of all relevant biases
**For data inputs that contribute to the analysis but were not synthesized as part of the study:**		
7	Describe and give sources for any other data inputs.	May score NOT RELEVANT
**For all data inputs:**		
8	Provide all data inputs in a file format from which data can be efficiently extracted (e.g. a spreadsheet rather than a PDF), including all relevant meta-data listed in item 5. For any data inputs that cannot be shared because of ethical or legal reasons, such as third-party ownership, provide a contact name or the name of the institution that retains the right to the data.	None
**Data analysis**		
9	Provide a conceptual overview of the data analysis method. A diagram may be helpful.	None
10	Provide a detailed description of all steps of the analysis, including mathematical formulae. This description should cover, as relevant, data cleaning, data pre-processing, data adjustments and weighting of data sources, and mathematical or statistical model(s).	None
11	Describe how candidate models were evaluated and how the final model(s) were selected.	None
12	Provide the results of an evaluation of model performance, if done, as well as the results of any relevant sensitivity analysis.	May score NOT RELEVANT if there were no results to publish
13	Describe methods for calculating uncertainty of the estimates. State which sources of uncertainty were, and were not, accounted for in the uncertainty analysis.	13a – describe the methods for calculating uncertainty 13b – state what the sources of uncertainty were
14	State how analytic or statistical source code used to generate estimates can be accessed.	None
**Results and discussion**		
15	Provide published estimates in a file format from which data can be efficiently extracted.	None
16	Report a quantitative measure of the uncertainty of the estimates (e.g. uncertainty intervals).	None
17	Interpret results in light of existing evidence. If updating a previous set of estimates, describe the reasons for changes in estimates.	None
18	Discuss limitations of the estimates. Include a discussion of any modelling assumptions or data limitations that affect interpretation of the estimates.	None

Articles were assessed for the presence of items listed on the GATHER checklist by two independent reviewers (MC and JH), with use of a form (Supplementary material 3) and a table of keywords designed to aid extraction (Supplementary material 4). The presence of an item on the checklist was marked as YES or NO, and in certain situations NOT RELEVANT was also assigned either if that item was deemed to be irrelevant to the methodology of that study or when it was unclear from the methodology whether the item should have been reported. Items 4b, 7, and 12 were assessed as NOT RELEVANT most frequently, meaning that the study did not specify whether there were *post* *hoc* exclusions (4b), did not use any data sources without modification (7), or did not state whether model performance or sensitivity analysis was done, and therefore there would be no results to publish (12) . However, for four studies [12–15] items 3 through to 5 were not relevant because these studies combined previously calculated health estimates.

### Analysis

In order to make an assessment of the compliance to the items of the GATHER checklist, the percentage of studies assigned YES, NO, and NOT RELEVANT was calculated per item. We then calculated a ‘GATHER performance score’ for each study. To avoid penalizing studies that didn’t report an item that was not required, the following equation was used to account for this, where a ‘YES’ scores 1 point, ‘NO’ scores 0, and ‘NOT RELEVANT’ takes 1 point away from the denominator (considering there were 21 items in our modified checklist):

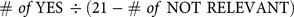

Finally, the GATHER performance scores were compared across study types and their characteristics.

## Results

### Search

The sensitivity of the final search terms (calculated as the percentage of target articles identified by the search) was 69% (18/26). The remaining target articles and the search were combined to give a total of 7,436 studies (Figure 1). Initial TIAB screening excluded 7,091 as either irrelevant or duplicates. The remaining 345 full texts were retrieved; 156 studies were excluded for ineligibility or if the full text could not be obtained. One hundred and eighty-nine journal articles remained; to this 23 UN reports were added. Thus 212 studies were eligible for full data extraction.

### Inter-observer agreement

Cohen’s kappa for inter-observer agreement was calculated for TIAB and full text inclusions and exclusions, where 0.7 was deemed the lowest acceptable value [16]. The agreement for the assessment of TIAB eligibility was 0.73, and the agreement for full text eligibility was 0.83.

### Descriptive characteristics

Table 2 gives the characteristics of the 212 studies included in the scoping review. One hundred and four (49%) of the studies made health estimates for the global population, 30 (14%) made estimates for multiple countries, and 78 (37%) made estimates at a national or subnational level. The majority of studies made estimates of health status (81%, 172 studies), with the remaining 35 (17%) studies making estimates of health determinants, and 5 (2%) making estimates of both. The most common country of origin for reports of health estimates was the USA (31%, 65 studies), followed by Switzerland (15%, 32 studies) and the UK (14%, 29 studies); studies from these countries were dominated by global or multinational studies. The remaining studies originated from 28 other countries, with 20 (9%) or fewer reports from each country; the majority of studies from these other countries were national or subnational in scope. All studies were published between 2010 and 2015. In total, there were 94 publishing journals. The most common journal was the *Lancet*, where 32 (15%) of the articles were published, followed by 23 (11%) in *PLOS One* and 23 (11%) UN reports. The remaining 134 articles (64%) were published by 91 other journals, each publishing 6 or fewer. Out of the articles published in journals, 52 articles (28%) were published in journals with impact factors greater than or equal to 10, and 137 (72%) were published in journals with impact factors of less than 10. Articles reporting global or multinational studies were more likely to be published in high-impact-factor journals (38% (43) of global/multinational articles were published in journals with impact factors greater than 10).

**Table 2.** Characteristics of the 212 studies included in the scoping review.

Category	Characteristic	Number out of *n* = 212 (%)
Country of origin according to the corresponding author	USA	65 (31%)
	Switzerland	32 (15%)
	UK	29 (14%)
	Australia	20 (9%)
	China	10 (5%)
	The Netherlands	7 (3%)
	Portugal	6 (3%)
	Other (≤ 5 publications, 24 countries)	43 (20%)
Year of publication	2010	17 (8%)
	2011	26 (12%)
	2012	45 (21%)
	2013	68 (32%)
	2014	51 (24%)
	2015	5 (2%)
Journal of publication	*Lancet*	32 (15%)
	*PLOS One*	23 (11%)
	UN report	23 (11%)
	*PLOS Medicine*	6 (3%)
	*BMC Public Health*	6 (3%)
	Other (≤ 5 publications, 90 journals)	122 (58%)
Impact factor	< 10	137 (64%)
	≥ 10	52 (25%)
Population studied	Global	104 (49%)
	Multi-country	30 (14%)
	National and subnational	78 (37%)
Indicator studied	Health status	172 (81%)
	Health determinant	35 (17%)
	Health status and determinant	5 (2%)

### State of GATHER reporting

**Figure 2.**
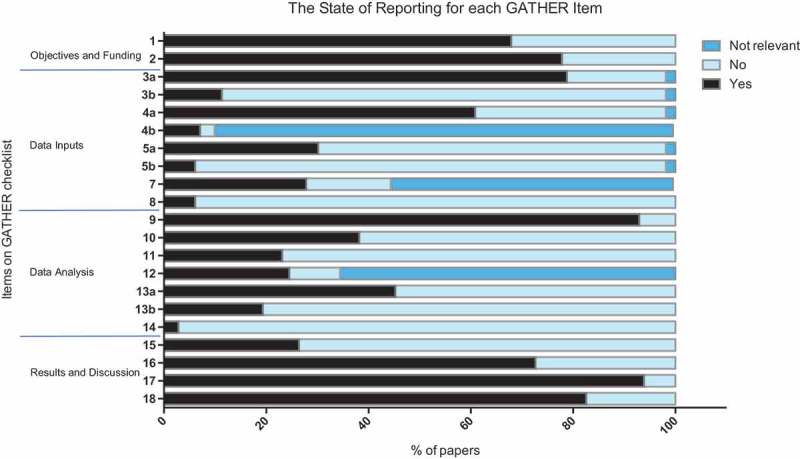
Stacked bar chart showing the percentage of studies that either correctly reported a GATHER item (reported), failed to report a GATHER item (not reported), or had methods that rendered a particular item irrelevant (not relevant). *n = *212.

Figure 2 shows the state of reporting for each individual item. The majority of studies defined the indicator of interest and the funding body (67.9 and 77.8% respectively for items 1 and 2). For the items categorized under data inputs, 78.8% of studies reported how they identified their data (3a), but only 11.3% described how it was accessed (3b); 60.8% of studies specified inclusion and exclusion criteria (4a), or the database used, and 7.1% of studies identified *post* *hoc* exclusions (4b); only 30.2% of studies reported the minimum of four characteristics of their included data sources (5a), with only 6.1% reporting the full seven characteristics (5b). 27.8% of studies described additional data sources (e.g. covariates; 7) and only 6.1% provided the data inputs in electronic format (8). For the data analysis reporting items, 92.9% of studies gave a conceptual overview of their method used to analyse data (9), but only 38.2% of studies provided a detailed description of the analysis, including mathematical formulae (10); additionally, only 23.1% of studies described how potential models were evaluated and the final model selected (11), and 24.5% published results of a model performance evaluation or sensitivity analysis (12). 45.3% described the methods used to calculate uncertainty estimates (13a), and 19.3% described the sources of uncertainty included and not included (13b); finally, only 2.8% of studies gave details on how to access the statistical code used (14). For items describing requirements for results and discussion sections, 26.4% of studies provided results in an electronic format (15); 72.6% of studies reported quantitative measures of uncertainty (16); 93.9% discussed their results in light of existing evidence (17); and 82.5% discussed the limitations of their methods and estimates (18).

The best-performing items were items 9 (a conceptual overview of the analysis) and 17 (discussing the results in light of existing evidence), with 92.9 and 93.9% of the studies reporting these two items respectively. Conversely, items on open access (5b, 8, and 14) were least likely to be reported, where only 6.1, 6.1, and 2.8% of studies reported these items respectively. It is worth noting that 4b (identifying any *post* *hoc* exclusions) appears to have a poor state of reporting; however, this is because for many of the studies 4b was not relevant. Of the 9.8% of studies where there were *post* *hoc* exclusions, 70% identified them. This is similar for items 7 (describing additional data sources) and 12 (reporting the results of a model evaluation or sensitivity analysis); the studies that described additional data sources (44.3%) and had results from a model evaluation or sensitivity analysis (34.4%) were more likely to report them than not (over 60% of the studies for which 7 and 12 were relevant reported them).

**Figure 3.**
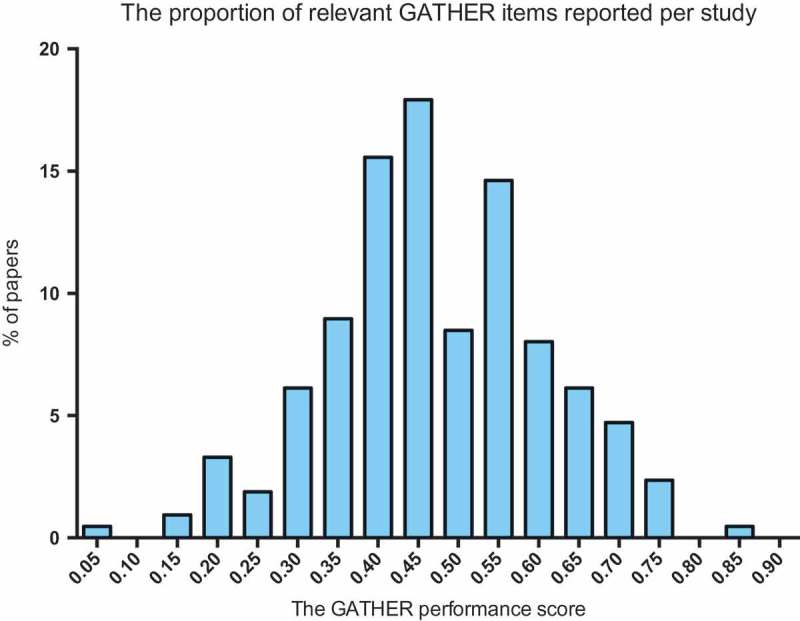
Bar chart depicting the distribution of the GATHER performance score across all papers, where the GATHER score is the proportion of relevant items being reported by a given study. *n *= 212.

**Figure 4.**
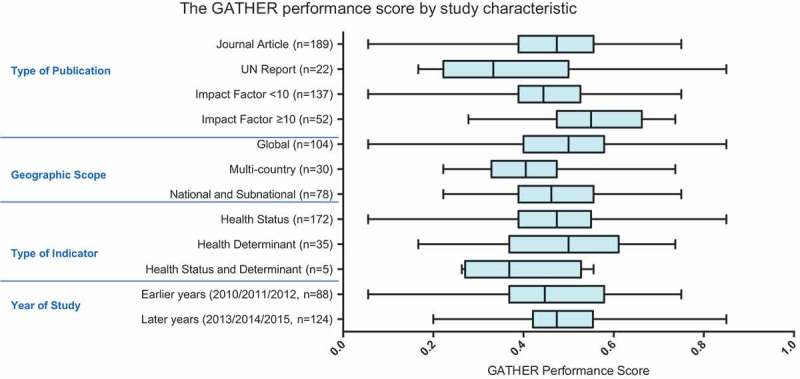
Box and whisker plot showing the GATHER performance score (the proportion of relevant items reported by a study) by study characteristics. Box depicts mean ± interquartile range, and whiskers the maximum and minimum values.

Figure 3 shows the distribution of the GATHER performance score across all studies. The highest-performing study, a UN report on maternal mortality [17], scored 0.85. The lowest-performing study scored 0.06, and the mean GATHER performance score was 0.47. Figure 4 shows the GATHER performance score by study characteristic. The mean GATHER performance score of articles published in journals with impact factors ≥ 10 was higher than the mean score of UN reports. However, there was no clear trend in GATHER performance score by geographic scope, year of publication, or whether the indicator study described health status, determinants, or both.

## Discussion

The data from this scoping review show that, on average, the frequency of reporting of the items on the GATHER checklist was middling. The mean GATHER performance score was 0.47, demonstrating that on average less than 50% of the items on the checklist were reported. The documentation of input data, availability of the input dataset, computer code, and results datasets were the most poorly reported. Additionally, information pertaining to all the steps involved in data analysis (mathematical formulae for all steps, model evaluation, and methods for uncertainty calculations) was also generally poorly reported, although most studies were able to provide a conceptual overview of the method used to analyse the data. Objectives of the study, funding, and discussions were usually well reported.

We assessed studies on the basis of information available in the journal article including appendices, or, in the case of UN reports, the web page on which the report is published. In certain cases, some of the authors were aware that additional information was available, but the reviewers were unable to locate it using the journal article or report (for example, the results were not on the webpage on which the report was found). In these cases, we considered that reporting was adequate only when information in the published article or report clearly guided the reader to the methods or data. Failure to identify the location of reporting items was a particular problem for UN reports, though it also occurred in journal articles. Thus, naming the location where each GATHER checklist item is reported will improve the access to important features of the study.

Moreover, we did not consider that citing another article or report for methods, a common practice, was sufficient for data analysis reporting items. This decision was made as (1) the citation then requires the reader to obtain a second article, which is not deemed to be ‘easily accessible’ information; and (2) the cited methodology may undergo changes in order to incorporate different input data. Without a detailed description of the method, it is difficult to make an assessment of whether there were any changes to the model which has been cited, hence making the methodology opaque.

Surprisingly, we found that 60.9% of studies did not make their results available in a format amenable to downstream research use, such as a spreadsheet. It is worth noting, for example, that many of the databases from UN reports are updated with newer estimates. This means that older estimates can no longer be easily accessed; we believe that older results should be archived. This may point to a wider problem, where there is no generalized method to archive output datasets for both journal articles and UN reports. We consider the failure to identify a permanent location for results to be a major deficiency in reporting – estimates have limited utility if users are unable to access or use them.

Likewise, the GATHER working group determined that availability of input datasets, computer code, and output datasets in machine-readable format is a part of minimum essential reporting, a recommendation that is unusual among reporting guidelines [19], but consistent with an increasing movement among funding agencies, journals, and experts on research integrity to maximize the utility of research by fully reporting data and methods [6,7,20–24]. Full transparency of data and methods – which in practice includes publication of datasets and computer code – is needed to allow other researchers to build upon published research and advance the science of health estimation. It may also increase confidence in published results by enabling external scrutiny of data and methods [25]. The primary obstacles to sharing of data and code are the professional structures in academia that reward publication of journal articles, but not underlying data and code, and that undervalue data management [20,21]. Therefore, to deal with this lack of access to input data, code, and results, we suggest that there is a need for the creation of incentive structures to reward transparent reporting within academia, such as the creation of data citation systems [26] and linking data citations to professional advancement.

Our review was subject to certain limitations. The authors who evaluated reporting in each study (MC and JH) did not have prior experience publishing global health estimates. Because the reviewers were not involved in any of the studies identified in the scoping review, they made an unbiased assessment of each study identified in the review. However, it was often challenging for the reviewers to determine whether some of the reporting items were met. For example, global health estimates often involve a sequence of analytic steps; the reviewers were unable to penalize authors who did not report some of the analytic steps, thus possibly overestimating whether a detailed description of all analytic steps was reported. Similarly, it was challenging for the reviewers to assess whether the authors described the limitations of their study accurately. For some reporting items, it was not always possible to determine whether reporting was omitted: for example, if an author did not report whether any *ad* *hoc* exclusions were made, or the author did not report whether model performance was evaluated. Future studies reporting in line with the GATHER recommendations will clearly state when these are not applicable. Finally, we targeted the review at studies assessing health indicators with a large burden or that were included in the MDGs, which may have had a different quality of reporting compared to studies of other health indicators in GATHER’s scope.

We conducted this scoping review in a similar manner to previously published studies assessing the quality of systematic reviews [18] or compliance to the QUOROM statement for meta-analyses [27]. These studies were conducted after the publication of the corresponding guidelines. By making an assessment of the quality of studies reporting health estimates *prior* to the publication of the GATHER guidelines, we have identified areas of the checklist that require particular attention. This should provide some additional guidance on such items when using GATHER, in conjunction with the example and explanation document that is published alongside the guidelines. Additionally, we have established the baseline quality of such studies. This may allow comparison to the state of reporting after the GATHER guidelines have been available for some time. It is the responsibility of all parties involved in producing health estimates – funding agencies, authors, editors, and reviewers – to ensure that the GATHER guidelines are implemented in order to ensure the improved transparency of health estimate reporting.

## Conclusions

It is evident that application of the GATHER guidelines will improve the quality of reporting of studies making health estimates, but that compliance will imply an additional workload for authors compared to current practices. Reports and journal articles publishing health estimates on average met less than half of the GATHER checklist items prior to the checklist’s publication. The items that were least likely to be met were on documentation of data sources used, access to datasets, access to computer code, and access to results in an easy-to-analyse format, as well as a complete description of all steps relating to data analysis. Whilst this study is limited by the fact that the reviewers were non-experts in the range of fields studied, this scoping review has allowed the identification of the current state of reporting of information relating to the data and methods used to generate health estimates. Thus, we have highlighted the areas of the GATHER checklist that require the most improvement, with the view that this will aid future users of the GATHER checklist. We hope that the GATHER guidelines will improve the reporting of health estimates, and additionally, may improve the reporting of studies that employ similar techniques but that are not strictly within the scope of GATHER.

## Paper context

Health estimates are increasingly useful to global health, but often employ complex methods. The Guidelines for Accurate and Transparent Health Estimates Reporting (GATHER) checklist describes 18 items to be reported in every study publishing health estimates, allowing users to assess methods. We performed a scoping review to establish the state of reporting in studies published prior to GATHER. Currently, the reporting of health estimate methodology is incomplete; the use of GATHER should improve this.
